# Coffee consumption is associated with a decreased risk of incident chronic kidney disease: A protocol for systematic review and meta-analysis

**DOI:** 10.1097/MD.0000000000027149

**Published:** 2021-09-17

**Authors:** Yudong Li, Wenchang Li, Yisheng Lu, Jing Zhang

**Affiliations:** a Department of General Medicine, Wuliqiao Community Health Center, Huangpu District, Shanghai, China; b Department of Internal Medicine, Shanghai Xinqidian Rehabilitation Hospital, Shanghai, China; c Department of Clinical Laboratory, Ruijin Hospital (Luwan Branch) of Shanghai Jiaotong University, School of Medicine, Shanghai, China.

**Keywords:** chronic kidney disease, coffee consumption, meta-analysis, systematic review

## Abstract

**Background:**

Recent studies have suggested a renal protective effect of coffee consumption against development of chronic kidney disease (CKD), although the results remain inconclusive. We performed a protocol for systematic review and meta-analysis to comprehensively investigate this association by summarizing all available data.

**Methods:**

An all-round retrieval will be performed in 5 electronic journal databases from their inception to June 2021, which comprise Medline, PubMed, Embase, ScienceDirect, and the Cochrane Library. The following key words were used on combination with Boolean operators AND or OR: “coffee,” “caffeine,” “renal insufficiency,” “chronic kidney diseases,” “chronic renal diseases.” Two authors completed the quality assessment using the Newcastle–Ottawa Scale for observational studies. The meta-analysis was conducted using Review Manager 5.3 software from the Cochrane Collaboration (London, UK).

**Results:**

The findings of this study will be submitted to peer-reviewed journals for publication.

**Conclusion:**

Coffee consumption may be associated with a lower risk of incident CKD.

## 1 Introduction

Chronic kidney disease (CKD) is a clinical syndrome secondary to the definitive change in function and/or structure of the kidney and is characterized by its irreversibility and slow and progressive evolution.^[1,2]^ It is most commonly attributed to diabetes and/or hypertension, but other causes such as glomerulonephritis, infection, and environmental exposures (such as air pollution, herbal remedies, and pesticides) are common in many developing countries.^[3–5]^ The prevalence of CKD is estimated to be 8% to 16% worldwide.^[6,7]^ It is emerging as a complex global health problem with a huge economic burden both on the affected family of patients and on the health care delivery system.^[8]^ Prevention of development and progression of CKD is, therefore, vital from public health perspective. Current recommendations focus on prevention and management of treatable causes and modifiable risk factors of CKD such as, diabetes mellitus, hypertension, and smoking, as well as use of angiotensin-converting enzyme inhibitors or angiotensin receptor blockers in selected patients.^[9]^

Coffee is one of the most popular consumed beverages in the world. Habitual coffee consumption may contribute favorably or harmfully to general health, systemic metabolism, and prevention or development of critical diseases such as cardiovascular disease and cancers.^[10,11]^ Such effects would be of great scientific interest and it is important to address the potential public health implications. Although the association between coffee consumption and risk of cardiovascular disease and hypertension remains inconclusive, coffee consumption has been found to be inversely associated with dementia, insulin resistance, type 2 diabetes mellitus, cirrhosis, and increased risk of osteoporosis.^[12,13]^ However, the effects of coffee on kidney function remain unclear. To summarize all existing data, this protocol for systematic review and meta-analysis was conducted by comprehensively identifying all cohort studies that compared the risk of incident CKD in coffee-drinkers versus nondrinkers and combining their results together.

## 2 Methods

### 2.1 Study registration

This protocol of systematic review and meta-analysis is based on the Preferred Reporting Items for Systematic Reviews and meta-analysis Protocols (PRISMA-P) statement guidelines.^[14]^ We have registered the protocol on the Open Science Framework (10.17605/OSF.IO/UJHCG). As this study is on the basis of published or registered studies, ethical approval and informed consent of patients are not required.

### 2.2 Search strategy

An all-round retrieval will be performed in 5 electronic journal databases from their inception to June 2021, which comprise Medline, PubMed, Embase, ScienceDirect, and the Cochrane Library. The following key words were used on combination with Boolean operators AND or OR: “coffee,” “caffeine,” “renal insufficiency,” “chronic kidney diseases,” “chronic renal diseases.” References of the included articles were also scanned for potentially relevant studies. No restrictions were placed on the publication language.

### 2.3 Inclusion and exclusion criteria

Studies that were eligible for the meta-analysis must be cohort study that investigated the incidence of CKD after index date between coffee-drinkers and non-drinkers. Participants with prevalent CKD before the start of the study (i.e., at the entry of cohort) must be excluded from the analysis. Eligible studies must report relative risk (RR), hazard ratio (HR) or standardized incidence ratio (SIR) with 95% confidence intervals (CIs) comparing the risk between the groups. Alternatively, the studies may provide sufficient raw data to calculate one of the aforementioned ratios. Publications in either full-length article or conference abstract format were eligible for inclusion if they provided sufficient information to satisfy the inclusion criteria.

### 2.4 Data extraction

A standardized data collection form was used to extract the following information: title of the study, first author's last name, journal where the study was published, year of publication, year(s) of conduct, country of origin, background study population, methods used to identify/enroll coffee-drinkers and non-drinkers, definition of coffee-drinkers, number and baseline characteristics of coffee-drinkers and non-drinkers, duration of follow-up, confounders that were adjusted in multivariate analysis, and adjusted effect estimates with 95% CI. To ensure the accuracy of the data extraction, both investigators independently reviewed and filled out the data collection forms for all studies, which were later cross-checked. Data discrepancy was caught and the original article of the data in question was jointly re-reviewed by both investigators.

### 2.5 Quality assessment

Two authors completed the quality assessment using the Newcastle–Ottawa Scale for observational studies.^[15]^ Items assessed included selection of cases/cohorts and controls, comparability of study design and analysis, outcome assessment, and adequacy of follow-up. “Stars” were allocated for each item included in the Newcastle–Ottawa Quality Assessment Scale for a quantitative appraisal of overall quality of the individual studies. A maximum of 9 stars can be allocated to any 1 study. A study was considered to have a low risk of bias if it was allocated the maximum number of stars. A median score of 6 stars was used to distinguish moderate-and high-quality studies from poorer-quality studies.

The evidence grade was assessed using the guidelines of the GRADE (Grading of Recommendations, Assessment, Development, and Evaluation) working group^[15]^ including the following items: risk of bias, inconsistency, indirectness, imprecision, and publication bias. GRADE pro Version 3.6 software is used for the evidence synthesis.

### 2.6 Statistical analysis

The meta-analysis was conducted using Review Manager 5.3 software from the Cochrane Collaboration (London, UK). Generic inverse-variance method as described by DerSimonian and Laird Point was used to pool effect estimates from all included studies.^[16]^ This technique assigns the weight for each study in the meta-analysis in reverse to its variance. As the assumption of fixed-effect model that all studies should give rise to the exactly same effect estimate is not true in most circumstances, especially for meta-analysis of observational studies that comprised of studies with different methodologies and background populations, random-effect model was used instead of fixed-effect model. Cochran Q test, which is complemented with the *I*^2^ statistic, was used to quantify the between-study statistical heterogeneity. A value of *I*^2^ of 0% to 25% represents insignificant heterogeneity, more than 25% but less than or equal to 50% represents low heterogeneity, more than 50% but less than or equal to 75% represents moderate heterogeneity, and more than 75% represents high heterogeneity.

## 3 Discussion

The current study is the first systematic review and meta-analysis to explore the risk of incident CKD among coffee-drinkers versus nondrinkers. The precise mechanism on how coffee consumption decreases the risk of renal impairment is a subject for debate.^[17]^ The most possible explanation is related to anti-oxidative effect of coffee, as atherosclerotic injury to the kidneys is one of the most common underlying mechanisms of development of CKD.^[10]^ Caffeine, hydroxychloroquine, quinides, niacin, and chlorogenic acid are known to be antioxidants in coffee.^[18]^ The antidiabetic effect of coffee may also help prevent the development of diabetic nephropathy. However, caffeine in coffee may interact with adenosine receptors, and interference with anti-inflammatory and glomerular hemodynamic effects of adenosine leads to proteinuria and glomerular remodeling and sclerosis.^[19]^ Further detailed studies are needed to confirm the current association and whether this effect of coffee consumption on glomerular filtration rate is beneficial for the kidney. A prospective study is also needed to examine the causality of this association.

## Author contributions

Jing Zhang: language edit and fund support

Wenchang Li: data analysis

Yisheng Lu: study design

Yudong Li: writing

**Conceptualization:** Wenchang Li.

**Data curation:** Wenchang Li.

**Funding acquisition:** Yudong Li.

**Investigation:** Yisheng Lu.

**Methodology:** Jing Zhang.

**Writing – original draft:** Yudong Li.
